# Lentiviral Transduction-based CRISPR/Cas9 Editing of *Schistosoma mansoni* Acetylcholinesterase

**DOI:** 10.2174/1389202924666230823094608

**Published:** 2023-11-22

**Authors:** Xiaofeng Du, Donald P. McManus, Juliet D. French, Haran Sivakumaran, Rebecca L. Johnston, Olga Kondrashova, Conor E. Fogarty, Malcolm K. Jones, Hong You

**Affiliations:** 1 Infection and Inflammation Program, QIMR Berghofer Medical Research Institute, Brisbane, Queensland, Australia;; 2 Faculty of Medicine, The University of Queensland, Brisbane, Queensland, Australia;; 3 Cancer Research Program, QIMR Berghofer Medical Research Institute, Brisbane, Queensland, Australia;; 4 Centre for Bioinnovation, University of the Sunshine Coast, Sunshine Coast, Queensland, Australia;; 5 School of Veterinary Science, The University of Queensland, Gatton, Queensland, Australia

**Keywords:** *Schistosoma mansoni*, lentiviral transduction, CRISPR/Cas9, genome editing, acetylcholinesterase, efficiency

## Abstract

**Background:**

Recent studies on CRISPR/Cas9-mediated gene editing in *Schistosoma mansoni* have shed new light on the study and control of this parasitic helminth. However, the gene editing efficiency in this parasite is modest.

**Methods:**

To improve the efficiency of CRISPR/Cas9 genome editing in schistosomes, we used lentivirus, which has been effectively used for gene editing in mammalian cells, to deliver plasmid DNA encoding Cas9 nuclease, a sgRNA targeting acetylcholinesterase (*SmAChE*) and a mCherry fluorescence marker into schistosomes.

**Results:**

MCherry fluorescence was observed in transduced eggs, schistosomula, and adult worms, indicating that the CRISPR components had been delivered into these parasite stages by lentivirus. In addition, clearly changed phenotypes were observed in *SmAChE*-edited parasites, including decreased *SmAChE* activity, reduced hatching ability of edited eggs, and altered behavior of miracidia hatched from edited eggs. Next-generation sequencing analysis demonstrated that the lentiviral transduction-based CRISPR/Cas9 gene modifications in *SmAChE*-edited schistosomes were homology-directed repair predominant but with much lower efficiency than that obtained using electroporation (data previously published by our laboratory) for the delivery of CRISPR components.

**Conclusion:**

Taken together, electroporation is more efficient than lentiviral transduction in the delivery of CRISPR/Cas9 into schistosomes for programmed genome editing. The exploration of tactics for enhancing CRISPR/Cas9 gene editing provides the basis for the future improvement of programmed genome editing in *S. mansoni*.

## 1 INTRODUCTION

Schistosomiasis remains the first on the scale of devastating parasitic helminth diseases and affects 250 million people in 74 countries [1-4]. The human infection starts when the free-living larval cercariae penetrate the skin and transform into schistosomula in the lung, burrow into the vasculature and develop into dimorphic sexual adults. The paired mature worms lay eggs, which lodge in host tissues and induce immunological reactions, resulting in inflammatory and obstructive diseases. The eggs are also released into faeces or urine (depending on the species), causing environmental contamination and transmission of these parasites *via* the snail host. Currently, no human vaccine is available, and the treatment of schistosomiasis only relies on a single drug, praziquantel (PZQ). The potential emergence of PZQ-resistant schistosomes is an ever-present concern [1]. Notably, complete genome sequences of three main schistosome species, including *Schistosoma japonicum* [5, 6], *S. mansoni* [7, 8] and *S. haematobium* [9, 10], have been generated. However, the major hurdle in mining the genome of schistosomes is the paucity of suitable tools to effectively modify critical genes in these parasites. To explore functions of unknown genes in schistosomes, previous studies have attempted post-transcriptional gene silencing but resulted in variable levels of efficacy, and the RNA interference outcomes were either transient or the inheritance of gene silencing was not fully penetrant [11-15].

CRISPR/Cas9, as a powerful gene editing tool, has been broadly applied in various organisms because of its potency, versatility, high efficiency, and specificity [16-26]. The feasibility of CRISPR/Cas9 genome editing has been demonstrated in *S. mansoni*. However, the Cas9-induced editing efficiency in this multicellular parasite is modest [26-28]. The main challenges in the efficient application of this novel technique for the study of the functional biology of schistosomes and other parasitic worms lie in enhancing gene mutation efficiency [29]. The complex morphology and life cycles inherent to schistosomes are major hurdles in the efficient application of CRISPR/Cas9 for functional biology studies. Tactics developed for CRISPR/Cas9 studies on the model worm *Caenorhabditis elegans* [21, 30-34], mammalian cells [35-39] and uni-cellular parasites [40-45] provide valuable information for exploring programmed genome editing in schistosomes [29].

To date, the most applied approaches for the delivery of CRISPR components into helminths include microinjection, which is used as a ‘gold standard’ for introducing CRISPR components into cells, lentiviral transduction [46] and electroporation. Electroporation has also been applied in schistosomes for post-transcriptional gene silencing [11-15], and the procedure has recently been employed in CRISPR/Cas9 studies with modest gene mutation efficiency [26-28]. Compared with electroporation, lentiviral transduction has exhibited superiority in the delivery of transgenes into human cells with relatively high efficiency and limited cell death [47, 48]. As one of the most common and effective techniques for delivering CRISPR/Cas9 in mammalian cells, lentiviral transduction has only been applied in one study for programmed genome editing in schistosomes. Ittiprasert *et al.* utilized lentivirus to deliver CRISPR/Cas9 to *S. mansoni* eggs targeting the gene encoding omega-1 ribonuclease (ω1), which is critical for T helper type 2 (Th2) polarization and granuloma formation. It was found that CRISPR/Cas9 induced ~4.5% non-homologous end joining (NHEJ) modifications and a 0.19% frequency of homology-directed repair (HDR) in the knock-in eggs. These gene modifications resulted in remarkably changed phenotypes in ω1-edited eggs [26].

Our previous studies in our laboratory have demonstrated the feasibility and efficiency of applying CRISPR/Cas9 for the editing of *SmAChE* (encoding *S. mansoni* acetylcholinesterase) using electroporation [27]. AChE, as a recognized anthelminthic target [49], is a pivotal component in the cholinergic system of adult schistosomes, playing essential roles in a number of critical activities, including worm muscle function, sexual maturation, the mating of mature adult worms, and the modulation of parasite glucose scavenging from mammalian host blood [50-54]. By using electroporation for the delivery of CRISPR components, You *et al.* found that the CRISPR/ Cas9-induced gene modifications in *SmAChE*-edited eggs were HDR (~ 0.12%) predominant and the gene editing induced significant phenotypic changes both *in vitro* and *in vivo* studies [27].

To further optimize the CRISPR/Cas9 genome editing in *S. mansoni* and also to compare the efficiency of lentiviral transduction and electroporation in CRISPR/Cas9 delivery, we employed lentiviral approach to deliver CRISPR components into different developmental stages of *S. mansoni* for editing of *SmAChE*. We transduced *S. mansoni* eggs, schistosomula and adult worms with infectious lentiviral particles generated by transfection of HEK293T cells with a reconstructed CRISPR-lentiviral vector encoding a mCherry fluorescence marker, Cas9 nuclease and a specific single guide (sgRNA) targeting the exon 5 of *SmAChE* [27]. The gene modification efficiency was then evaluated using NGS and monitoring resulting phenotypic changes.

## 2 MATERIALS AND METHODS

### 2.1 Parasites

*S. mansoni* cercariae were obtained by shedding infected *Biomphalaria glabrata* snails under bright light. Female Swiss mice (six weeks old) were infected with 100 *S. mansoni* cercariae subcutaneously and euthanized seven weeks post-infection. *S. mansoni* adult worms were recovered by portal perfusion with 37°C pre-warmed RPMI medium 1640 (Gibco, Sydney, Australia) and cultured in RPMI complete medium (RPMI Medium 1640 (Gibco) plus 100 IU/ml penicillin and 100 μg/ml streptomycin (Gibco) 10% (v/v) and heat-inactivated fetal bovine serum (FBS, Gibco)) at 37°C in 5% CO_2_. *S. mansoni* liver eggs were isolated from infected mouse livers as described [55] and cultured in RPMI complete medium. Mature and immature liver eggs were separated as described [56]. Miracidia were harvested by hatching eggs in deionized water under light [57]. Schistosomula were obtained by mechanical transformation of cercariae *in vitro* and cultured in Basch’s medium as described [57, 58]. Eggs were collected from *S. mansoni* adult worms *in vitro* cultured for 24 h and named Day1 eggs.

### 2.2 SgRNA Design and Plasmid Reconstruction

**Fig. (1):**
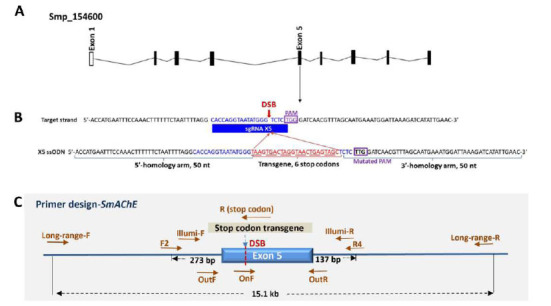
Genomic structure of the site encoding *SmAChE* in the genome of *S. mansoni* and the design for CRISPR/Cas9-mediated editing of *SmAChE*. (**A**) Gene model of *SmAChE* (Smp_154600) showing the position of its exons and introns. (**B**) *SmAChE* exon 5 illustrating the location and sequence of the sgRNA X5 target locus, predicted double-strand break (DSB) (red arrow), PAM (TGG, purple box), and 124-nucleotide sequence of X5ssODN that was provided for DNA repair by HDR. The PAM sequence in the donor template was mutated to TTG. Homology arms of 50 nt flank a central 24 nt of a six-stop-codon transgene. The six-stop codon in X5ssODN is predicted to be inserted into the DSB during the repair of DSB by HDR. (**C**) Schematic diagram showing binding sites and directionality of each primer (brown arrows). The forward primer F2 paired with a reverse primer R, which is specific for the 6-stop codon transgene and covers 310 bp around the target site, was employed to detect the integration of transgene at the predicted DSB. The primer pair F2+R4 was used to generate control PCR amplicons (430 bp). Primer pairs OnF+OutR and OutF+OutR were designed for evaluating CRISPR/Cas9 gene editing efficiency at the X5 site using quantitative real-time PCR. Illumi-F and Illumi-R were utilized to amplify target amplicons (474 bp) for the construction of the next-generation sequencing (NGS) library. Primers Long-range-F and Long-range-R, covering 15.1 kb around the predicted DSB, were used for generating PCR amplicons for long-range PCR NGS.

SgRNAs were designed with online tools http://bioinfogp.cnb.csic.es/tools/breakingcas/ [59] and the Benchling software platform https://benchling.com, where predicted cleavage sites for the *Streptococcus pyogenes* Cas9 nuclease and double-strand breaks (DSBs) were identified within the genome of *S. mansoni*. The sgRNA X5 (5’-CACCAGGTAATATGGGTCTC-3’) targeted residues 722-741 in exon 5 (named X5) [27] of *SmAChE* (Smp_154600, Fig. **1A**), and these were followed by a *protospacer-adjacent motif* (PAM) sequence ‘TGG’ (Fig. **1B**). A specific X5 single-stranded oligodeoxynucleotide (X5ssODN) donor template, with homology arms of 50 nt flanking a central 24 nt of a six-stop-codon transgene (5'-TAAGTGACTAGG TAACTGAGTAGC-3'), was designed as a donor template for DNA repair by HDR (Fig. **1B**). The PAM sequence in the donor template was mutated to protect the HDR donor DNA from Cas9 cleavage. A non-targeting sgRNA (5'-GACCAGGATGGGCACCACCC-3') was used as negative control (NC). SgRNA X5 and X5ssODN were obtained from Integrated DNA technologies (Singapore).

SgRNAs were individually inserted into a LentiCRISPRv2-mCherry vector (a gift from Agata Smogorzewska, Addgene plasmid #99154, Addgene, Watertown, Massachusetts, USA) as described [60, 61]. Sanger sequencing was used to confirm the successful plasmid reconstruction using primer LKO.1 5' (oligo sequences are listed in Table **S1**). The reconstructed LentiCRISPRv2-mCherry vector encodes the *Streptococcus pyogenes* Cas9 nuclease driven by the human elongation factor-1 alpha (EF-1α) promoter, a mCherry red fluorescent marker and specific sgRNA driven by the human U6 promoter.

### 2.3 Production of Infectious Lentiviral Particles

HEK293T cells were cultured in RPMI complete medium at 37°C in 5% CO_2_. When cell growth reached 70-80% confluence, cells were transfected with the reconstructed LentiCRISPRv2-mCherry vector and two additional vectors: lentiviral packaging plasmid (pCMV-dR8.91, Addgene) that expresses HIV structural and packaging genes and envelope plasmid (pCMV-VSVG, Addgene) that expresses the pseudotyping envelope protein Vesicular Stomatitis Virus Glycoprotein (VSVG). Sixteen hours later, the media was removed from the transfected cells and replaced with 5% RPMI complete medium (RPMI Medium supplemented with 5% heat-inactivated FBS and 100 IU/ml penicillin and 100 μg/ml streptomycin). After 24 h, the supernatant containing lentiviral particles was collected and filtered through 0.45 µm pore size membranes and stored at 4°C (the first collection). An additional fresh 5% RPMI complete medium was added to the cell culture flask and cultured for another 24 h. The supernatant was collected as described above (the second collection) and then combined with the supernatant in the first collection and concentrated using a Lenti-X concentrator (Takara, Melbourne, Australia). Virion titer was measured by Lenti-X-GoStix (Takara) to establish the presence of functional virions at > 5 x 10^5^ infective units per ml (IFU/ml). The generated lentiviral particles were aliquoted and stored at -80°C before use.

### 2.4 Delivery of CRISPR Components into Schistosomes by Lentiviral Transduction

The *SmAChE* X5 site was targeted in *S. mansoni* eggs, schistosomula and adult worms. Given that previous studies have shown that targeting immature eggs may induce higher gene editing efficiency [62], we employed two populations of eggs for the gene editing, including liver eggs, which were a mix of mature and immature eggs and Day1 immature eggs, which were laid by adult worms after being *in vitro* cultured for 1 day. Pools of 10 000 liver eggs or 2000 Day1 eggs or 2000 schistosomula, or 5 pairs of adult worms were cultured with lentiviral particles (> 5 x 10^5^ IFU/ml) at 37°C in 5% CO_2_ for 18 h. Polybrene (Hexadimethrine bromide, Sigma-Aldrich, Sydney, Australia) (8 μg/ml) was added to the transduction of eggs. For target-specific knock-in (KI), parasites were washed with PBS to remove lentiviral particles 18 h after co-culturing and then transfected with specific X5ssODN (6 µg) (X5-KI) by square wave electroporation in a chilled 0.4 cm Gene Pulser Electroporation Cuvette (Bio-rad, Sydney, Australia) in 200 µl Opti-MEM medium (Gibco) with a 20 ms pulse at 125 V. Treated parasites were cultured in RPMI complete culture medium at 37°C in 5% CO_2_ for two days. Wild-type (WT) parasites and parasites transduced with negative control lentiviral particles (Con) and transfected with/without X5ssODN and parasites transfected with X5ssODN in the absence of virions served as negative controls. *SmAChE*-edited and control liver eggs and Day1 eggs were cultured for 7 days when all eggs were considered to be mature [63] and were then hatched to miracidia. The egg-hatching efficiency was evaluated through dividing the number of hatched eggs by the total number of examined eggs X 100%.

### 2.5 Detection of mCherry Fluorescence in Transduced Schistosomes

Transduced eggs, schistosomula, and adult worms from each group were fixed in 1 ml 10% formalin for at least 1 h and then stained with 1 µg/ml DAPI (4, 6-diamidino-2-phenylindole) (Sigma-Aldrich) at room temperature for 15 min. Stained parasites were washed three times in PBS and then transferred to a slide with Aqueous Mounting Medium (Sigma-Aldrich) for examining mCherry red fluorescence under a Zeiss 780 NLO confocal microscope (Zeiss, Oberkochen, Germany).

### 2.6 PCR Amplification of Transgene to Detect KI at Target Locus

PCR assays were performed using template genomic DNA generated from CRISPR/Cas9-edited parasites and the transgene-specific primers to reveal the integration of transgene at the target site. The genomic DNA of parasites was extracted using the E.Z.N.A. tissue DNA kit (Omega BIO-TEK, Norcross, Georgia, USA) according to the manufacturer’s instructions. Each genomic DNA sample was subjected to two PCR assays using two distinct primer pairs. One primer pair included a reverse primer named R, specific for the stop-codon transgene in X5ssODN, pairing with a forward primer termed F2 (Fig. **1C**, Table **S1**). The other primer pair, composed of a reverse primer R4 and a forward primer F2, was used to generate positive control amplicons (Fig. **1C**, Table **S1**). The PCR reaction mixture included a 12.5 µl Green GoTaq DNA polymerase mix (Promega) with 400 nM of each primer and 30 ng genomic DNA. Thermal cycling conditions involved denaturation at 95°C for 3 min, followed by 40 cycles of 95°C, 30 s, 58°C, 30 s and 72°C, 1 min and a final extension at 72°C for 5 min.

### 2.7 *SmAChE* Activity Assay

The enzymatic activity of *SmAChE* in *SmAChE*-edited schistosome developmental stages was measured to assess the gene editing efficiency at the protein level. *S. mansoni* soluble egg antigen (SEA) preparation, soluble worm antigen preparation (SWAP) and soluble schistosomula antigen were generated as described [64, 65]. The *SmAChE* activity in SWAP (7.5 μg/ml), SEA (0.45 μg/ml) and soluble schistosomula antigen (0.45 μg/ml) were determined using an Amplex Red Acetylcholine/Acetylcholinesterase Assay Kit (Invitrogen, Melbourne, Australia) as per the manufacturer’s instructions.

### 2.8 Miracidial Behavioral Assays

To determine whether the gene editing could be delivered from eggs to miracidia and to compare the gene mutation efficiency in *SmAChE*-edited mature eggs and immature eggs, *S. mansoni* liver eggs were separated into mature and immature eggs [56] before being subjected to gene editing. Eggs were then cultured in RPMI complete medium for 7 days and hatched to miracidia. The behavior of miracidia hatched from *SmAChE*-edited mature and immature liver eggs was monitored as described. Briefly, ~30 miracidia in 100 μl deionized water was transferred to the centre of a glass microscope slide. The miracidia swimming (movement) pattern was monitored using an Olympus-CKX41 microscope with a DP22 Digital Microscope Camera (Olympus, Shinjuku City, Tokyo, Japan). Miracidial movement in the field of view (FOV) was recorded by video for 1 min. The recorded miracidial videos were analyzed using FIJI software as described [66]. Using TrackMate plugin, miracidia locations along an x-y axis were tracked in each frame and then combined into complete tracks for each individual miracidium showing in the FOV. The MTrackJ plugin was employed to determine three behavioral measures: duration (time) of miracidia presence in the FOV, tortuosity (reflecting the magnitude and frequency of circling and turning) of miracidial movement [67] and average velocity of miracidial swimming. Heatmaps, representing the movement pattern of individual miracidia, were generated as described [68, 69]. Five repeats were performed for each group.

### 2.9 Real-time PCR for Quantifying Gene Editing Efficiency

Genomic DNA extracted from *SmAChE*-edited parasites was used for two independent quantitative real-time PCR (qPCR) assays using two pairs of primers (‘ON’ primer pair and ‘OUT’ primer pair) (Fig. **1C**, Table **S1**) for evaluating gene editing efficiency as described. The ‘OUT’ (flanking) primer pair (OutF+OutR) was used as a control for normalization purposes and was designed with at least 50 bp surrounding the sgRNA binding region. The ‘ON’ (overlapping) (OnF+OutR) included one OnF primer that bound the 20 bp of the sgRNA target sequence and a reverse OutR primer. This approach uses the fact that gene mutation occurring at a target site would affect the binding of the OnF primers, leading to delayed real-time PCR amplification and higher quantification cycle (Cq), while the binding of the ‘OUT’ primers is not affected. Accordingly, the efficiency of mutagenesis can be determined by comparison of the ON/OUT Cq ratios of edited and non-edited samples [70, 71].

The qPCR was conducted using QuantiNova SYBR^®^ Green PCR Kits (Qiagen) using a Corbett Rotor-Gene 6000 Real-Time PCR system (Qiagen). Each qPCR reaction incorporated 10 µl 2xSYBR Green PCR Master Mix (Qiagen), 30 ng genomic DNA, and 0.7 µM of each primer. The cycling parameters were set as follows: 95°C for 5 min, 40 cycles of 95°C for 30 s, 58°C for 30 s and 72°C for 30 s.

### 2.10 Illumina Sequencing and CRISPResso2 Data Analysis

NEBNext Ultra II Q5 Master Mix (New England Biolabs, Ipswich, MA, USA) was used to generate PCR amplicons (474 bp) for Illumina Sequencing using the MiSeq primer pair (Illumi-F and Illumi-R) (Fig. **1C**, Table **S1**) and pooled genomic DNA samples extracted from experimental groups. Each PCR reaction mix (50 µl) contained 25 µl NEBNext Ultra II Q5 Master Mix, 60 ng genomic DNA, 1 µM of each primer, and H_2_O. The PCR program was set as follows: 98°C for 30 s, 35 cycles of 98°C for 10 s, 64°C for 30 s and 72°C for 30 s, and a final extension for 2 min at 72°C. PCR amplicons were purified using a QIAquick PCR Purification Kit (Qiagen). PCR products produced from four independent PCR reactions from each sample were pooled, and 100 ng of PCR amplicons from each group were utilized to generate the uniquely indexed paired-end read libraries. Then, these libraries were pooled, quantified using an Agilent 2100 bioanalyzer (Agilent Technologies, Santa Clara, USA), and subjected to sequencing on the MiSeq platform using 300 bp paired-end reads (Illumina, San Diego, CA, USA).

After sequencing, raw fastq files from each individual sample were analyzed to determine on-target gene editing activities utilizing CRISPResso2 (v2.0.30) [72]. CRISPResso2 parameters were set, as previously reported by our laboratory. Importantly, a 1 bp window was used to avoid PCR and/or sequencing artifacts being detected as false positive non-homologous end joining (NHEJ) indels or substitutions. A custom Python script was used to analyze potential HDR reads as described. The HDR reads reported by CRISPResso2 were further confirmed with Fasta36 sequence comparison software (https://github.com/wrpearson/fasta36) [27]. These filtered reads were utilized to calculate the frequencies of NHEJ and HDR events.

### 2.11 Long-range PCR Next-generation Sequencing and Data Analysis

To test the possibility of large deletions in modified parasites, we conducted long-range PCR NGS, which relied on high-throughput sequencing of long-range PCR amplicons of up to 20 kb in size [73, 74]. Genomic DNA from each individual *SmAChE*-edited male and female worm was used as a template to generate long-range PCR amplifications utilizing the primers, Long-range-F, and Long-range-R (Fig. **1C**, Table **S1**), covering 15.1 kb around the predicted DSB and PrimeSTAR GXL DNA Polymerase (Takara, Melbourne, Australia), according to the manufacturer’s protocols. The PCR reaction mixture comprised 10 μl 5X PrimeSTAR GXL Buffer, 4 μl dNTP Mixture (2.5 mM each), 1 μl PrimeSTAR GXL DNA Polymerase, 0.2 μM of each primer, 35 ng genomic DNA, and sterile purified water to 50 μl. The PCR reaction parameters were set as 30 cycles of 98°C 10 s and 68°C 10 min. The long-range PCR amplicons were virtualized and confirmed on an agarose gel and then purified using ExoSAP-IT™ PCR Product Cleanup Reagent (Thermo Fisher Scientific). All amplicons were quantified using a Qubit dsDNA HS Assay Kit (Invitrogen), and 1 ng of each sample was used for the construction of each NGS library with Nextera*^®^* XT DNA Sample Preparation Kit (Illumina), according to the manufacturer’s instructions. Prepared libraries were quantified using an Agilent 2100 bioanalyzer and then sequenced on the MiSeq platform (Illumina).

The analysis of long-range PCR NGS data involved manual inspection of the bam files. Samtools version 1.9 (https://github.com/samtools/samtools) was used to extract all reads that map to *SmAChE* (Smp_154600) exon 5 (coordinates SM_V7_1:88640405-88640645) and calculate the number of correctly paired reads. Samclip version 0.4.0 (https://github.com/tseemann/samclip) was used to extract all soft-clipped reads that mapped to *SmAChE* exon 5. These reads were also manually inspected using Integrative Genomics Viewer (IGV) version 2.5.0.

### 2.12 Statistical Analysis

All data are shown as the mean ± SE. Differences between groups were determined for statistical significance by One-way ANOVA and where appropriate, by two-tailed Student’s t-test. GraphPad Prism software (Version 8.2.1, La Jolla, CA, USA) was used for all statistical analyses. A statistically significant difference for a particular comparison was defined as a *p*-value ≤ 0.05. * *p* value≤ 0.05, ** *p* value≤ 0.01, *** *p* value ≤ 0.001, **** *p* value ≤ 0.0001, not significant (ns).

## 3 RESULTS

### 3.1 MCherry Fluorescence in Transduced Schistosomes

**Fig. (2):**
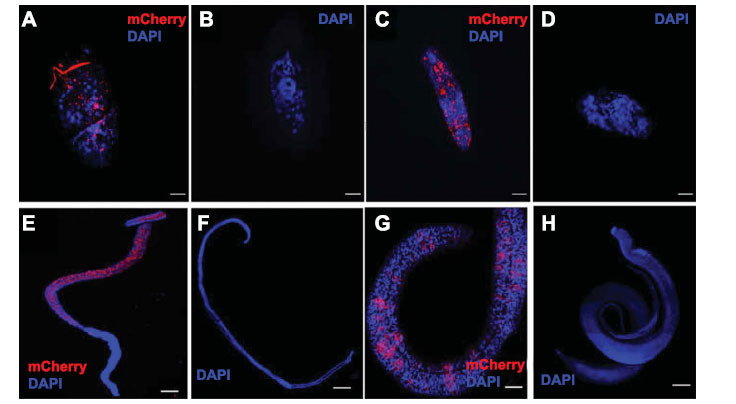
Confocal microscopy images of *S. mansoni* parasites transduced with lentiviral particles. MCherry fluorescence (red) was observed in transduced (**A**) egg, (**C**) schistosomulum, (**E**) adult female worm, and (**G**) adult male worm. Wild-type (WT) (**B**) egg, (**D**) schistosomulum, (**F**) female worm, and (**H**) male worm were used as controls. All samples were DAPI-stained (blue). Scale bars in A-D = 20 µm; Scale bars in E-H = 200 µm.

Lentiviral particles (> 5 x 10^5^ infective units per ml, Fig. **S1**) were used to transduce *S. mansoni* developmental stages for programmed editing of *SmAChE* (Fig. **2**). The mCherry red fluorescence was observed in *S. mansoni* eggs (Fig. **2A**), schistosomula (Fig. **2C**), adult female worms (Fig. **2E**) and adult male worms (Fig. **2G**) transduced with lentiviral particles, while no mCherry fluorescence was detected in untreated WT eggs (Fig. **2B**), schistosomula (Fig. **2D**), female adults (Fig. **2F**), and adult males (Fig. **2H**), indicating the successful delivery of CRISPR components into transduced parasites.

### 3.2 Detection of Integration of Transgene at Target Site by PCR Assays

**Fig. (3):**
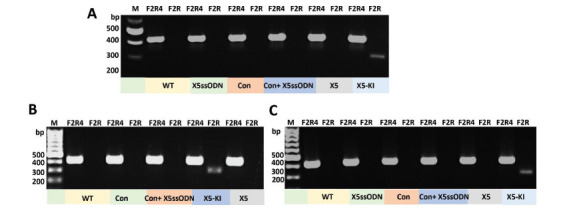
PCR assays demonstrating CRISPR/Cas9-mediated integration of the transgene in exon 5 of *SmAChE*. (**A**) Genomic DNA extracted from WT liver eggs and liver eggs treated with X5ssODN, Con (negative control lentiviral particles), Con+X5ssODN, X5 (X5 lentiviral particles), and X5-KI (X5 and X5ssODN) were used as the PCR templates. (**B**) Genomic DNA extracted from miracidia hatched from WT liver eggs and liver eggs treated with Con, Con+X5ssODN, X5-KI, and X5 were subjected to PCR assays. (**C**) Genomic DNA extracted from WT schistosomula and schistosomula treated with X5ssODN, Con, Con+X5ssODN, X5, and X5-KI were used as PCR templates. Positive control PCR amplicons (430 bp) generated with the F2+R4 primers were observed in all samples. Evidence of integration of transgene at the target site X5 was revealed by PCR products amplified with the F2+R primers, and these were only observed in parasites treated with X5-KI, with the expected size of 307 bp.

Considering that the X5ssODN donor DNA template includes a 6-stop codon transgene, which facilitates genotyping [27], PCR assays were conducted using template genomic DNA extracted from the *SmAChE*-edited eggs, miracidia hatched from *SmAChE*-edited eggs, and *SmAChE*-edited schistosomula to detect the site-specific KI. Each genomic DNA sample was subjected to two independent PCR assays using two distinct primer pairs comprising F2+R, which is specific for the six-stop-codon transgene and F2+R4, which served as a positive control (Fig. **1C** and Table **S1**). PCR amplicons generated with the F2+R4 primers (430 bp) were observed in all samples (Fig. **3**). The transgene-specific PCR products amplified with the F2+R primers with the expected size of 307 bp were only observed in X5-KI parasites, indicating the successful KI of the transgene at the X5 target site of *SmAChE* in these samples (Fig. **3**).

### 3.3 Quantification of Gene Modification Using Real-time PCR Assays

**Fig. (4):**
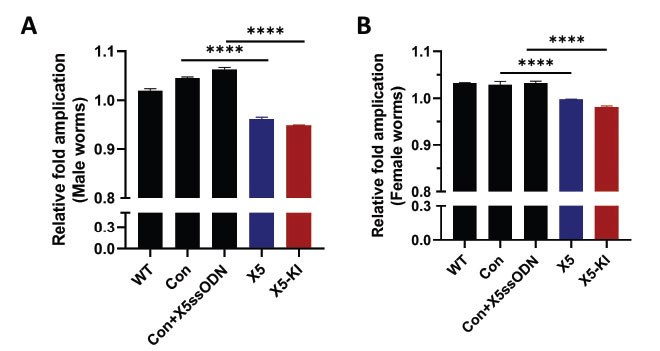
Quantification of CRISPR/Cas9 gene editing efficiency using real-time PCR assays. (**A**) Template genomic DNA extracted from WT adult male worms and male worms treated with Con, Con+X5ssODN, X5, and X5-KI were used for real-time PCR assays. (**B**) Template genomic DNA from WT adult female worms and female worms treated with Con, Con+X5ssODN, X5, and X5-KI were used for real-time PCR assay. (**** *p* value≤ 0.0001, One-way ANOVA).

To estimate the gene editing efficiency at the target locus, we performed quantitative real-time PCR assays using template genomic DNA extracted from *SmAChE*-edited adult male and female worms [70, 71]. We found that the relative fold amplification in male worms treated with X5 and X5-KI was reduced by 4% (*p*<0.0001) and 5.2% (*p*<0.0001), respectively (Fig. **4A**), and in female worms treated with X5 and X5-KI, it was 0.25% (*p*<0.0001) and 2% (*p*<0.0001), respectively (Fig. **4B**).

### 3.4 Detection of CRISPR/Cas9-induced HDR and NHEJ in *SmAChE*-edited Parasites

**Fig. (5):**
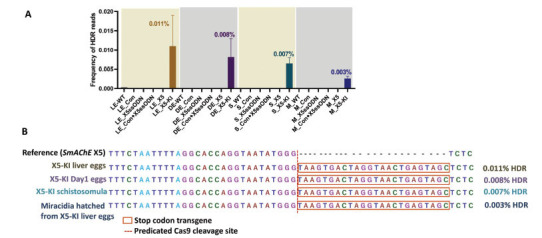
Frequency of HDR reads in *SmAChE*-edited schistosomes. (**A**) Next-generation sequencing (NGS) revealed HDR efficiency in X5-KI treated liver eggs (LE), Day1 eggs (DE), schistosomula (S) and miracidia (M) hatched from X5-KI treated liver eggs. WT parasites and parasites treated with Con, X5ssODN, Con+X5ssODN or X5 were used as controls. HDR reads were determined with CRISPResso2 and confirmed utilizing fasta36. (**B**) Sequence alignments and confirmed HDR reads in each KI group. The nucleotide sequence for *SmAChE* exon 5 (X5) and the predicted Cas9 cleavage site were demonstrated. At the predicted cleavage locus, the integration of a 24 nt stop codon transgene (orange box) was confirmed. The number of confirmed HDR reads and HDR frequencies, representative of a percentage of aligned reads, are shown to the right of each group. Two biological repeats were used for X5 and X5+X5ssODN groups.

An amplicon NGS approach was employed to examine the HDR and NHEJ efficiency in *SmAChE*-edited *S. mansoni* developmental stages. Genomic DNAs extracted from *SmAChE*-edited liver eggs, *SmAChE*-edited Day1 eggs, *SmAChE*-edited schistosomula, and miracidia hatched from *SmAChE*-edited liver eggs were used for sequencing. Barcoded amplicon libraries were constructed from pooled genomic DNA of parasites exposed to sgRNA X5 and X5ssODN. The sequencing was conducted on the Illumina MiSeq platform, and deep-coverage sequence reads were analyzed using CRISPResso2 [72]. On average, 245 600 sequenced reads per sample were aligned to the reference amplicon sequence of the Smp_154600 locus. Using a window width of 1 bp, which limits the number of PCR and/or sequencing errors detected as NHEJ reads, we found that the NHEJ frequency in *SmAChE*-edited parasites was similar to that in control parasites (WT, Con, X5ssODN and Con+X5ssODN) (Table **S2**). The confirmed HDR frequency in X5-KI treated liver eggs, X5-KI Day1 eggs, and X5-KI schistosomula was 0.011%, 0.008%, and 0.007%, respectively (Fig. **5**, Table **S2**). In addition, a very rare HDR frequency (0.003%) was also detected in miracidia hatched from X5-KI treated liver eggs (Fig. **5**, Table **S2**). Two biological repeats were used for *SmAChE*-edited groups.

### 3.5 Decreased Hatching Ability of *SmAChE*-edited *S. mansoni* Eggs

**Fig. (6):**
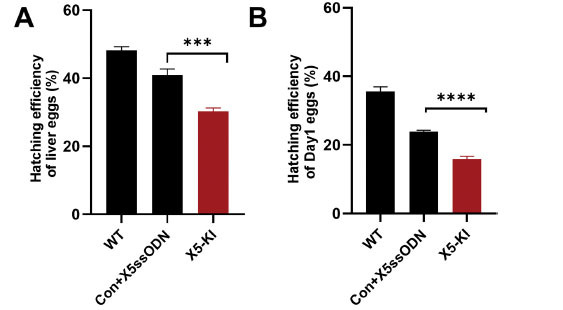
Reduced hatching ability of *SmAChE*-edited *S. mansoni* eggs. (**A**) Hatching efficiency of WT liver eggs and liver eggs treated with Con+X5ssODN and X5-KI. (**B**) WT Day1 eggs and Day1 eggs treated with Con+X5ssODN and X5-KI were used for hatching. (*** *p*-value ≤ 0.001, One-way ANOVA).

To determine the effect of gene editing on eggs, we examined the hatching ability of *SmAChE*-edited liver eggs and Day1 eggs. Significantly declined hatching efficiency was observed in X5-KI treated liver eggs (26.1%, *p*=0.0002) (Fig. **6A**) and X5-KI treated Day1 eggs (34.2%, *p*<0.001) (Fig. **6B**). In addition, we recorded a reduction in hatching efficiency in both Con+X5ssODN treated liver eggs (*p*=0.0011) and Day1 eggs (*p*<0.001) compared with WT liver eggs and WT Day1 eggs, respectively, indicating the treatment of Con and X5ssODN likely influenced egg hatching (Fig. **6**).

### 3.6 Behavioral Modifications in Miracidia Hatched from *SmAChE*-edited *S. mansoni* Mature and Immature Liver Eggs

**Fig. (7):**
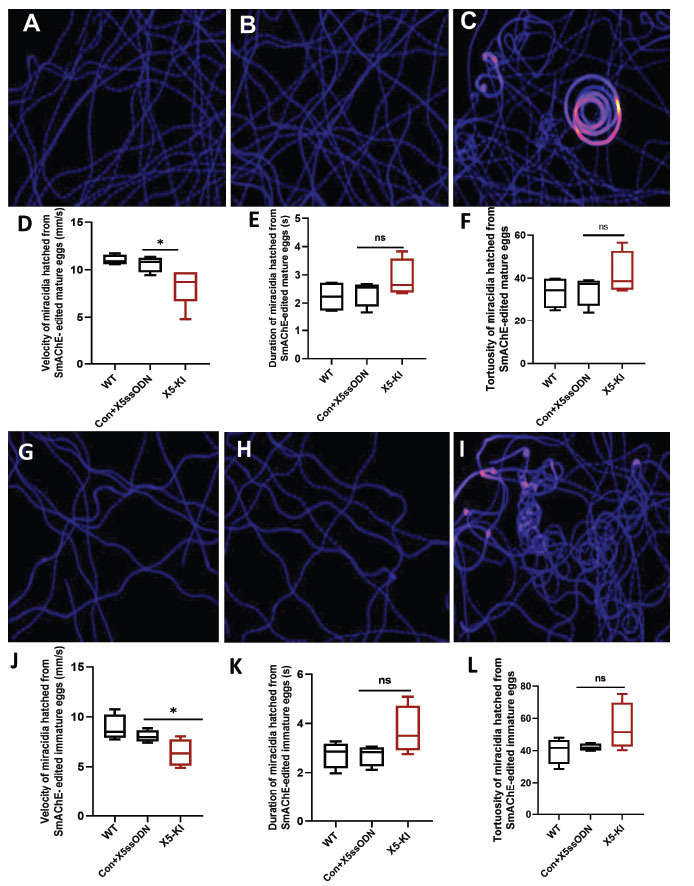
Behavioral modifications of *S. mansoni* miracidia hatched from *SmAChE*-edited mature and immature liver eggs. Heatmaps (**A**-**C**) representative of the swimming patterns of individual miracidia hatched from WT mature liver eggs and mature liver eggs treated with Con+X5ssODN and X5-KI, respectively, within a 1 min recording. Colors in these heatmaps indicate the time of miracidia spent in a specific site. Black: absence; Blue: shorter time presence; Red and Yellow: longer time presence. Boxplots demonstrating swimming (**D**) velocity, (**E**) duration and **(F**) tortuosity of miracidia hatched from WT mature liver eggs and mature eggs treated with Con+X5ssODN and X5-KI. Heatmaps (**G**-**I**) showing the swimming patterns of individual miracidia hatched from WT immature liver eggs and immature liver eggs treated with Con+X5ssODN and X5-KI, respectively. The moving (**J**) velocity, (**K**) duration, and (**L**) tortuosity of miracidia collected from WT immature eggs and immature eggs treated with Con+X5ssODN and X5-KI were determined. (ns - not significant; * *p* value≤ 0.05, two-tailed t-test).

To further compare the gene editing efficiency in mature and immature eggs, we performed CRISPR/Cas9 using mature and immature liver eggs and hatched these eggs to miracidia after culturing for 7 days when all eggs were considered to be mature [63]. The behavior of miracidia hatched from *SmAChE*-edited mature (Fig. **7A**-**F**) and immature eggs (Fig. **7G**-**L**) was examined by analyzing the recorded videos of miracidial movement. Heatmaps were generated showing the movement patterns of individual miracidia within the 1 min recording. For the miracidia hatched from mature liver eggs, the heatmaps of the control miracidia, including miracidia hatched from WT mature liver eggs (Fig. **7A**) and mature liver eggs treated with Con+X5ssODN (Fig. **7B**), demonstrated linear soft blue lines. In contrast, more abundant red and yellow regions were observed in the heatmaps of miracidia hatched from X5-KI mature eggs (Fig. **7C**), indicating relatively slower movement and more turning and circling behavior of these miracidia in the field of view (FOV). The swimming velocity of miracidia hatched from X5-KI mature eggs was markedly decreased by 21.6% (*p*=0.01) compared with miracidia hatched from Con+X5ssODN-treated mature eggs (Fig. **7D**). The moving duration and tortuosity of miracidia hatched from X5-KI mature eggs were slightly but not significantly affected (Fig. **7E**-**F**).

Similarly, heatmaps, representative of the swimming patterns of individual miracidia hatched from X5-KI immature liver eggs (Fig. **7I**), demonstrated a relatively longer presence and more turning movement of these miracidia in the FOV, compared with the heatmaps of miracidia hatched from control groups [WT immature liver eggs (Fig. **7G**) and Con+X5ssODN-treated immature liver eggs (Fig. **7H**)]. The swimming velocity of miracidia collected from X5-KI immature eggs was clearly reduced by 20.2% (*p*=0.0344) compared with those hatched from Con+X5ssODN-treated immature eggs (Fig. **7J**). Furthermore, slightly but not significantly enhanced swimming duration and tortuosity were evident in miracidia hatched from X5-KI immature eggs (Fig. **7K**-**L**).

### 3.7 Decreased *SmAChE* Enzymatic Activity in *SmAChE*-edited Parasites

**Fig. (8):**
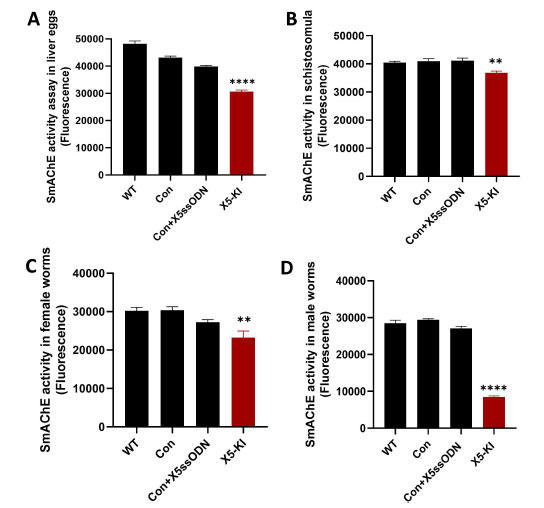
Reduced AChE enzymatic activity in *SmAChE*-edited *S. mansoni* parasites. (**A**) AChE activity in soluble egg antigen (SEA) extracted from WT liver eggs and liver eggs treated with Con, Con+X5ssODN, X5 or X5-KI. (**B**) Soluble schistosomula native antigens extracted from WT schistosomula and schistosomula treated with Con, Con+X5ssODN, X5 or X5-KI were used for AChE activity assays. (**C**) AChE activity in soluble worm antigen preparation (SWAP) produced from WT female worms and female worms treated with Con, Con+X5ssODN, X5 or X5-KI. (**D**) AChE activity in SWAP generated from WT male worms and male worms treated with Con, Con+X5ssODN, X5 and X5-KI. (* *p* value≤ 0.05, ** *p* value≤ 0.01, **** *p* value ≤ 0.0001, One-way ANOVA).

To further investigate the CRISPR/Cas9 editing of *SmAChE* at the translational level, we performed *SmAChE* activity assays. The enzymatic activity of *SmAChE* was considerably decreased by 25.6% (*p*<0.0001) and 10.3% (*p*=0.0013) in X5-KI treated liver eggs (Fig. **8A**) and X5-KI schistosomula (Fig. **8B**), respectively. Similarly, reduced *SmAChE* activity was also observed in X5-KI-treated female worms (14.7%, *p*=0.003) (Fig. **8C**) and X5-KI-treated male worms (68.8%, *p*<0.0001) (Fig. **8D**).

### 3.8 Investigation of the Potential of Large Deletions at the Target Site

To further investigate the causes of the significant phenotype changes observed in *SmAChE*-edited parasites, we performed long-range PCR NGS to determine whether large deletions occurred at the target site, as reported in the parasitic nematode *Strongyloides stercoralis* [24]. Genomic DNA extracted from individual X5-KI or controls (WT and Con+X5ssODN treated) adult *S. mansoni* was used to generate long-range PCR amplicons using a primer pair Long-range-F and Long-range-R, which covers 15.1 kb around the DSB (Fig. **1C**, Table **S1**). Sequencing data were analyzed using coordinates SM_V7_1:88640405-88640645 to extract reads that mapped to *SmAChE* exon 5. Based on these coordinates, an average of 99.88% of reads were properly mapped and paired (range 99.79-99.93%) across all samples (including controls), indicating very few discordant read pairs. Additionally, no mapped patterns of soft-clipping (which would indicate large deletions) were observed at the start or end of these extracted reads. These results showed no patterns that could corroborate the capture of potential large deletions by the long-range PCR NGS.

## 4 DISCUSSION

Delivery of CRISPR components remains an important factor in fully realizing the potential of CRISPR/Cas9 in gene editing. This is especially the case with parasitic worms, such as the schistosomes, which possess a complex morphology and have a number of different developmental stages during their life cycle [29]. Here, we discussed the possibility and drawbacks of employing lentiviral transduction to deliver CRISPR/Cas9 into schistosomes, an approach that has been shown to provide a higher level of efficiency than electroporation in human cells [47, 48]. We also demonstrated the potential and challenges of using a fluorescence marker to enrich transduced schistosomes and monitored differences in gene editing efficiency in schistosome developmental stages.

We transduced *S. mansoni* eggs, schistosomula and adult worms with prepared lentiviral virions carrying CRISPR/ Cas9 components targeting the X5 site of *SmAChE*. The mCherry red fluorescence was detected in transduced schistosomes, suggesting that the lentiviral transduction had successfully delivered the CRISPR/Cas9 into these parasites. However, harvesting mCherry- positive schistosomes is challenging. This is mainly because a large number of eggs (10 000/well) and schistosomula (2000/well) were required to perform downstream genomic sequencing and phenotypic analysis; furthermore, these parasites are too large and/or fragile to be sorted using conventional flow cytometry [29]. Alternative selectable markers developed for other organisms, such as the drug resistance marker selected and the co-CRISPR strategy [30, 31, 40, 75-79], may also be considered for enriching schistosome parasites with CRISPR components before further analysis. Given the difficulties in collecting and recovering transduced (mCherry-positive) schistosomes, we used pooled samples for downstream sequencing and phenotypic analysis. NGS using PCR amplicons with 474 bp spanning, the predicted DSB, and subsequent sequencing data analysis and utilizing a 1 bp window showed low frequency (≤0.011%) of HDR events occurred at the target site, and no NHEJ induced indels (small insertions and deletions) or substitutions were detected. This HDR-predominant DNA repair feature was supported by a previous study in our laboratory [27]. Notably, the HDR was also detected in miracidia hatched from *SmAChE*-KI eggs, albeit in low efficiency, indicating the possibility of applying the CRISPR/Cas9 gene editing to establish a transgenic line of schistosomes.

In addition, we employed two populations of eggs for gene editing: liver eggs (a mixture of mature and immature eggs) and Day1 eggs (immature eggs), as previous studies had shown that targeting immature eggs may induce higher gene modification efficiency [62]. However, a similar level of HDR frequency and phenotypic change (decreased egg hatching efficiency) were detected in *SmAChE*-edited liver eggs and *SmAChE*-edited Day1 eggs. Furthermore, we found that the behavioral changes of miracidia hatched from *SmAChE*-edited mature liver eggs and immature liver eggs were also comparable, indicating a similar level of gene modification induced by lentiviral delivery in both mature and immature eggs. In addition, we found that the efficiency of lentiviral transduction-based CRISPR/Cas9-induced gene editing in *SmAChE*-edited eggs in the current study was much lower than that was previously reported by Ittiprasert *et al.* [26]. This might be explained as follows: (i). Different vectors were used to express CRISPR components, which may have resulted in differing amounts of sgRNA and Cas9 in transduced parasites. (ii). The function and/or distribution of the targeted gene may also affect gene editing efficiency. (iii). In the NGS data analysis, a different quantification window size parameter, which defines the size (in bp) of the quantification window extending from the DSB, was used for the detection of the modifications. Ittiprasert *et al.* utilized a 202 bp window size [26], whereas we used a 1 bp window size to limit the amount of PCR and/or sequencing errors from being inappropriately quantified as modified reads or false positive results. Our previous study, where we ran CRISPResso2 with increasing window size parameters (values of 1, 20, 100, and 0), demonstrated that the numbers of NHEJ modifications predominated with NHEJ substitutions (>95%) and consistently increased across all negative controls and experimental samples, indicating the possibility of generating false positive [61].

The apparent paradox between the rare gene editing efficiency and the strong phenotypes observed in this study was also reported in previous CRISPR/Cas9 studies on *S. mansoni* [26, 27]. To further identify whether large deletions may have occurred and caused the strong phenotypes in CRISPR/Cas9 edited schistosomes, as previously observed in *S. stercoralis* [24], we performed long-range PCR NGS using amplicons covering 15.1 kb surrounding the Cas9 cleavage site. However, no large deletions were confirmed in *SmAChE*-edited schistosomes. An alternative explanation for the strong phenotypes may be that knocking down of one key gene, even in low efficiency in the whole parasite, may up- or down-regulate the transcription of other relative genes resulting in the significant phenotype changes, thereby indirectly amplifying the effect of the CRISPR/Cas9 mediated editing. Another possible reason for the marked phenotypic changes in this study may be the lentiviral vector delivered Cas9 that persistently bound the target site after cleavage, which blocked access to the DSB by DNA repair enzymes, rendering reduced DNA repair efficiency, as reported in mammalian cells [80]. The persistent Cas9 binding at the target site may affect target gene functioning, thereby resulting in phenotypic changes. Also, off-target events may occur [46] when using lentiviral transduction to deliver CRISPR/Cas9 into schistosomes. This might be because lentiviral vectors can provide a sustained expression of Cas9 in transduced cells, which may facilitate off-target Cas9 cleavages [46, 81]. Indeed, it has been reported that an important element affecting the number of off-target modifications is the amount and persistence of Cas9 expression in target cells, where high amounts of Cas9 increase non-specific nuclease cleavage and lower amounts of nuclease enhance the precision of cleavage [81, 82]. Moreover, we cannot exclude the possibility of more complicated DNA DSB repair events (*e.g*., chromosomal rearrangements) that cannot be detected by the methodologies utilized here. Further work will likely be required to characterize the mechanisms for DNA DSB repair in schistosomes.

Other non-viral delivery methods promoted for nematodes and mammalian cells may also guide further improvement in the delivery of CRISPR into schistosomes. Microinjection and the newly developed lipofection-based microinjection [83] have achieved high gene editing efficiency in the model worm *C. elegans* and other nematodes [21, 33, 83]. Also, the cell-penetrating peptide-mediated method [38, 84, 85] can rapidly internalize components across biological membranes both *in vivo* and *in vitro*. Additionally, the lipid nanoparticles (LNP)-mediated delivery system, which is biodegradable and well-tolerated, has high efficiency with minimal off-targets [86-88]. Using plasmid DNA instead of ssODN as a donor template [89] may improve the precise genome editing in schistosomes, a strategy that has been successfully employed in *S. stercoralis* for significantly increased HDR frequency [24]. Moreover, using chemicals, such as RS-1, which has also been reported to enhance the HDR efficiency in mammalian cells [90, 91], may also be feasible to enhance precision genome manipulation in schistosomes. Further optimization of CRISPR genome editing in schistosomes may also consider using alternative Cas9 orthologous [92-95] or different Cas proteins [96-98]. In addition, using a synthesized Cas9-sgRNA ribonucleoprotein (RNP) complex combined with a non-viral delivery method may foster an approach to enhance gene editing efficiency while minimizing off-target events [99]. Another interesting future direction is to consider establishing stable schistosome stem cell lines [100], whereby the CRISPR-mediated gene functional studies can be performed on a single-cell level so that the difficulties in applying CRISPR caused by the complex morphology may be bypassed.

## CONCLUSION

Overall, the outcomes from this study showed that lentiviral transduction could deliver CRISPR components into schistosome parasites, as evidenced by mCherry fluorescence observed in CRISPR-transduced parasites, albeit with limited gene editing efficiency. The complexity of the schistosomes may render difficulties in detecting gene mutations and obtaining an ideal level of gene editing efficiency, as has been reported in *S. stercoralis* [24]. Together with our previous studies [27], it is clear that electroporation is more efficient (10x) than lentiviral transduction in the delivery of CRISPR components into schistosomes for programmed genome editing. Further studies are required in order to further optimize the CRISPR/Cas9 work pipeline to fulfill the potential of this powerful gene editing tool for future study of schistosomes.

## Data Availability

The original contributions presented in the study are included in the article. Further inquiries can be directed to the corresponding author.

## LIST OF ABBREVIATIONS

DSBs: Double-strand Breaks
EF-1α: Elongation Factor-1 alpha
FOV: Field of View
HDR: Homology-directed Repair
IGV: Integrative Genomics Viewer
KI: Knock-In
LNP: Lipid Nanoparticles
NC: Negative Control
NHEJ: Non-homologous End Joining
PAM: Protospacer-adjacent Motif
PZQ: Praziquantel
SEA: Soluble Egg Antigen
SWAP: Soluble Worm Antigen Preparation
Th2: T Helper Type 2
VSVG: Vesicular Stomatitis Virus Glycoprotein
WT: Wild-type
