# Supplementary material for: Lentiviral Transduction-based CRISPR/Cas9 Editing of Schistosoma mansoni Acetylcholinesterase
Source: Curr Genomics. 2023 Nov 22;24(3):155–70. doi: 10.2174/1389202924666230823094608 (PMC10761339; doi:10.2174/1389202924666230823094608)
Supplement: Supplementary file 1 [file CG-24-155_SD1.pdf]

Supplementary Material

Lentiviral Transduction-based CRISPR/Cas9 Editing of *Schistosoma mansoni* Acetylcholinesterase

Xiaofeng Du[1,2], Donald P. McManus[1,2,†], Juliet D. French[3], Haran Sivakumaran[3], Rebecca L. Johnston[3], Olga Kondrashova[3], Conor E. Fogarty[4], Malcolm K. Jones[5] and Hong You[1,5,*]

[1]Infection and Inflammation Program, QIMR Berghofer Medical Research Institute, Brisbane, Queensland, Australia; [2]Faculty of Medicine, The University of Queensland, Brisbane, Queensland, Australia; [3]Cancer Research Program, QIMR Berghofer Medical Research Institute, Brisbane, Queensland, Australia; [4]Centre for Bioinnovation, University of the Sunshine Coast, Sunshine Coast, Queensland, Australia; [5]School of Veterinary Science, The University of Queensland, Gatton, Queensland, Australia

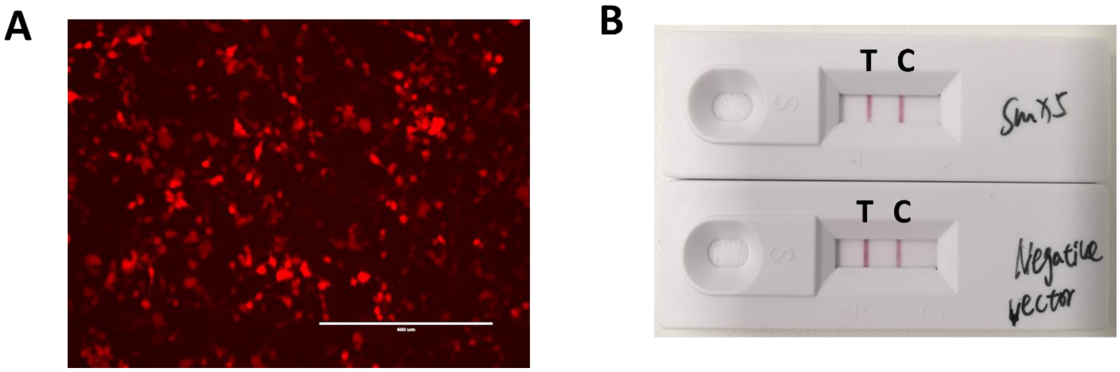

Figure S1 Generation of lentiviral particles by transfection of HEK293T cells. (**A**) MCherry fluorescence (in red) in transfected HEK 293T cells under fluorescent microscopy. Scale bars = 400 µm. (**B**) Measurement of virion titers of prepared lentiviral particles targeting X5 site (top cassette) and negative control lentiviral particles (bottom cassette). 'C' is the positive control band. The 'T' band indicates the sample has more than 5 x 10^5 infective units per ml (IFU/ml).

Table S1. Sequences of oligonucleotides.

Name	Sequence (5'-3')
LKO.1 5'	GACTATCATATGCTTACCGT
F2	TTTCAGGTAGTTCATAATGCAATCA
R	GCTACTCAGTTACCTAGTCACTTA
R4	TGAGACCATGGACTGACGAC
OnF	CACCAGGTAATATGGGTC
OutF	TTTAGTGTTGTAAATCAAATCTACTCCA
OutR	TGATACGATATGGATCACCACCAA
Illumi-F	ATCATTCAACGGAACGATTTGGG
Illumi-R	ATATGATTGTGCAAAAGAGGGACAT
2kb-F	TTATGGATATACGGTGGTAGCTTTTATATGG
2kb-R	ATACTGATTGGCTACTTCTTTGGTTTTATAGGC
Long-range-F	ATGCTCGGTAACTTGACCAGCGTAGCTTCT
Long-range-R	TGGTAAACCACCATCACCATGTTGGTTTTG

Table S2. Frequency of gene modification in next generation sequencing data analyzed using CRISPResso2.

Groups	Total_ reads	Aligned_ reads	Unmodi- fied	NHEJ	NHEJ_ Inser- tions	NHEJ_ Dele- tions	NHEJ_ Substitu- tions	HDR	Fil- tered_ reads	Filtered_ reads _percent	Unmodi- fied_ percent	NHEJ_ percent	NHEJ_ Insertions _percent	NHEJ_ Deletions _percent	NHEJ_Substitut ions _percent	HDR_ percent	HDR_ con- firmed
LE (liver eggs)-WT	253439	238821	229883	209	0	5	204	1	230093	96.34538	99.90873	0.090832837	0	0.002173034	0.088659803	0.000435	1
LE-X5ssODN	263955	253352	243711	215	0	0	215	0	243926	96.27948	99.91186	0.088141486	0	0	0.088141486	0	0
LE-Con	248966	241438	232171	210	0	3	207	0	232381	96.24873	99.90963	0.090368834	0	0.001290983	0.089077851	0	0
LE-Con+X5ssODN	239075	231747	222732	189	0	4	185	0	222921	96.19154	99.91522	0.084783399	0	0.001794358	0.082989041	0	0
LE-X5	284108	275944	265071	208	0	3	205	0	265279	96.13509	99.92159	0.078408016	0	0.001130885	0.077277131	0	0
LE-X5-repeat	284108	275944	265071	208	0	3	205	0	265279	96.13509	99.92159	0.078408	0	0.001131	0.077277	0	0
LE-X5+X5ssODN	272344	263289	253118	213	0	3	210	47	253378	96.2357	99.89739	0.084064126	0	0.001184002	0.082880124	0.018549	47
LE-X5+X5ssODN-repeat	267045	251066	241384	245	1	9	235	6	241635	96.24362	99.89612	0.101393	0.000414	0.003725	0.097254	0.002483	6
DE (Day1 egg)-WT	288178	270468	260067	237	0	5	232	1	260305	96.24244	99.90857	0.091047041	0	0.001920824	0.089126217	0.000384	0
DE-X5ssODN	289569	275973	264973	245	0	8	237	0	265218	96.10288	99.90762	0.092376837	0	0.003016387	0.089360451	0	0
DE-Con	263721	249501	239464	199	0	6	193	0	239663	96.05693	99.91697	0.083033259	0	0.002503515	0.080529744	0	0
DE-Con+X5ssODN	279509	267235	256601	218	0	3	215	0	256819	96.10231	99.91512	0.084884685	0	0.001168138	0.083716547	0	0
DE-X5	274604	260373	249804	215	0	3	212	0	250019	96.0234	99.91401	0.085993464	0	0.001199909	0.084793556	0	0
DE-X5-repeat	257086	244799	235268	172	0	1	171	0	235440	96.17686	99.92695	0.073055	0	0.000425	0.07263	0	0
DE-X5+X5ssODN	267765	253705	243382	228	0	0	228	31	243641	96.03319	99.8937	0.093580309	0	0	0.093580309	0.012724	31
DE-X5+X5ssODN-repeat	292029	274815	263841	260	1	6	253	9	264110	96.10465	99.89815	0.098444	0.000379	0.002272	0.095793	0.003408	9
S (schisto-somula)-WT	251556	247003	237116	237	0	4	233	0	237353	96.09316	99.90015	0.099851276	0	0.001685254	0.098166023	0	0
S-X5ssODN	249166	244156	234542	244	0	2	242	0	234786	96.16229	99.89608	0.103924425	0	0.00085184	0.103072585	0	0
S-Con	248229	243910	234294	199	1	5	193	0	234493	96.13915	99.91514	0.084863941	0.000426452	0.00213226	0.082305229	0	0
S-Con+X5ssODN	217254	212484	203918	243	0	1	242	0	204161	96.083	99.88098	0.119023712	0	0.00048981	0.118533902	0	0
S-X5	270254	266022	255240	225	3	6	216	0	255465	96.03153	99.91193	0.088074687	0.001174329	0.002348658	0.0845517	0	0
S-X5-repeat	254549	247012	237325	209	0	5	204	1	237535	96.16334	99.91159	0.087987	0	0.002105	0.085882	0.000421	1

S-X5+X5ssODN	243675	236508	226871	219	1	3	215	18	227108	96.0255	99.89564	0.096429892	0.000440319	0.001320957	0.094668616	0.007926	18
S-X5+X5ssODN-repeat	267369	263464	252874	219	1	6	212	12	253105	96.06815	99.90873	0.086525	0.000395	0.002371	0.08376	0.004741	12
M (miracid-ia)-WT (hatched from LE-WT)	203733	196974	189151	182	0	7	175	0	189333	96.12081	99.90387	0.09612693	0	0.00369719	0.09242974	0	0
M-X5ssODN (hatched from LE-X5ssODN)	264106	253324	242773	214	0	3	211	0	242987	95.91945	99.91193	0.088070555	0	0.001234634	0.086835921	0	0
M-Con (hatched from LE-Con)	239162	229422	220539	197	0	17	180	0	220736	96.21396	99.91075	0.089246883	0	0.007701508	0.081545375	0	0
M-Con+X5ssODN (hatched from LE-Con+X5ssODN)	217024	209644	201407	166	1	4	161	0	201573	96.15014	99.91765	0.082352299	0.000496098	0.001984393	0.079871808	0	0
M-X5 (hatched from LE-X5)	238944	228703	219614	177	0	1	176	0	219791	96.10324	99.91947	0.08053105	0	0.000454978	0.080076072	0	0
M-X5-repeat	229102	220685	211903	198	1	3	194	1	212102	96.11075	99.90618	0.093351	0.000471	0.001414	0.091465	0.000471	0
M-X5+X5ssODN (hatched from LE-X5+X5ssODN)	244978	231016	221802	210	0	4	206	7	222019	96.10546	99.90226	0.094586499	0	0.001801648	0.092784852	0.003153	7
M-X5+X5ssODN-repeat	245816	236334	226971	218	0	2	215	4	227193	96.13217	99.90229	0.095954	0	0.00088	0.094633	0.001761	4
